# iStent® Trabecular Microbypass Stent: An Update

**DOI:** 10.1155/2016/2731856

**Published:** 2016-06-20

**Authors:** Arthur Fernandes Resende, Neal Sanjay Patel, Michael Waisbourd, L. Jay Katz

**Affiliations:** Glaucoma Research Center, Wills Eye Hospital, Philadelphia, PA 19107, USA

## Abstract

Due to the high rates of complications and failure experienced with current glaucoma procedures, there is a continuous search for a safer and more effective glaucoma surgery. A new class of procedures termed minimally invasive glaucoma surgeries (MIGS) aim to fill this void by offering an alternative method of IOP reduction associated with markedly reduced complication rates and shorter recovery times. The iStent, a trabecular microbypass stent, is a MIGS device that has quickly gained popularity. The device allows aqueous humor to directly drain from the anterior chamber into Schlemm's canal by bypassing an obstructed trabecular meshwork. This review examines publications about the iStent, focusing on the device's efficacy, safety, and cost when a single iStent or multiple iStents are implanted in combination with cataract surgery or as a solo procedure. Current data suggest that the iStent is a safe and effective tool in the management of mild-to-moderate glaucoma, notable for its limited complications and absence of serious adverse events following implantation. As valuable experience is gained performing* ab interno* MIGS, increasing familiarity with angle anatomy and iStent placement, and as newer stent designs are developed, there is promise of continual improvement in the surgical management of glaucoma.

## 1. Introduction

Glaucoma is the leading cause of irreversible blindness worldwide, affecting over 65 million people [1]. The primary goal in the treatment of glaucoma is the management of intraocular pressure (IOP), which is traditionally first attempted through use of topical medications or laser therapy [2]. However, when these methods fail, surgery is often required to prevent vision loss. Due to the high rates of complications and failure experienced with current glaucoma procedures (e.g., trabeculectomy and tube shunt implantation) [3], there is a continuous search for a safer and more effective glaucoma surgery. Through the use of novel nonpenetrating and bleb-independent approaches, a new class of procedures termed minimally invasive glaucoma surgeries (MIGS) aim to fill this void by offering an alternative method of IOP reduction associated with markedly reduced complication rates and shorter recovery times compared with traditional glaucoma surgery [4–6].

**Figure 1:**
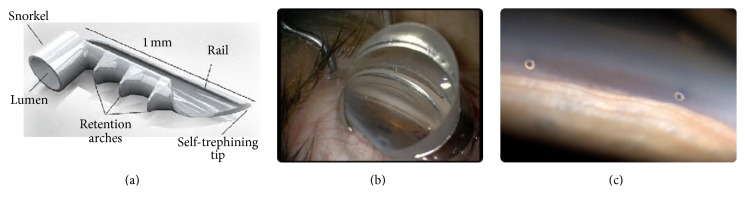
Illustration of the iStent with dimensions and technical specifications (a); intrasurgical view of the trabecular meshwork with a direct gonioscopy lens (b); flipped view of 2 inserted iStents under gonioscopy (c). ((a) and (b), courtesy of Glaukos Corporation; (c), courtesy of Matt Poe, http://www.ophthalmicphotography.info/).

The iStent (Glaukos, Laguna Hills, CA), a trabecular microbypass stent, is a MIGS device that has quickly gained popularity since first being published by Spiegel et al. in 2007 [7]. Being the smallest US Food and Drug Administration approved device ever implanted in the human body, the iStent is a 1 mm heparin-coated, nonferromagnetic, surgical grade titanium stent with a ridged, snorkel design pictured in Figure 1(a). The device allows aqueous humor to directly drain from the anterior chamber into Schlemm's canal by bypassing an obstructed trabecular meshwork. Requiring only a short surgical procedure for implantation, the iStent benefits from a relatively fast learning curve; it is inserted* ab interno* through a clear corneal incision guided by direct gonioscopy (Figure 1(b)). Additionally, the iStent has the potential to be a fiscally favorable alternative to traditional treatments by reducing medication burden in the long term [8]. Herein, we review the literature on the iStent trabecular microbypass stent published between January 2007 and April 2016 in order to better understand its efficacy, safety, cost considerations, and future directions. A PubMed search for “iStent” revealed 44 articles. Each of these full-text articles was reviewed. Secondary searches for “trabecular bypass” and minimally invasive glaucoma surgery or “MIGS” identified additional relevant articles. Randomized controlled trials (RCT) and relevant case series were included in this review and are listed in Tables 1 and 2. Review articles and cost studies were cited in this paper as well.

## 2. Outcomes of iStent Implantation with Cataract Surgery

Although cataract surgery is known to reduce IOP by itself (by approximately 2 mmHg) [9], combining this procedure with the implantation of an iStent through the same surgical incision can have a greater impact on reducing IOP and medication burden [10–12]. As such, the most popular and well-researched use of the iStent is when its implantation is performed simultaneously with cataract surgery. One of the earliest reports of this combined surgery in 2008 [13] demonstrated that 70% of subjects (*n* = 33/47) were able to discontinue all previous IOP lowering medications, with a mean IOP reduction of 5.7 ± 3.8 mmHg at 6 months (25.4% reduction, *P* < 0.001), a reduction greater than what has been evidenced in previous studies with cataract surgery as a solo procedure [10, 12–15].

A 2013 prospective, uncontrolled, interventional case series by Patel et al. [14, 16] examined the efficacy and outcomes of the combined iStent implantation and cataract surgery in 40 eyes with open-angle glaucoma (OAG). The study concluded that the procedure resulted in a significant reduction in IOP at 6 months postoperatively, with a mean reduction of 4.4 mmHg from a mean baseline IOP of 21.1 mmHg (20.9% reduction, *P* < 0.0001). Dependence on topical IOP lowering medication was also reduced significantly, with a mean number of medications reduced from 2.3 to 0.6 (*P* < 0.01). By a 6-month follow-up, 66% percent of patients were medication-free, with further 20% only requiring 1 ocular hypotensive medication; additionally all patients on oral acetazolamide prior to surgery (*n* = 6) were able to discontinue its use.

An identical case series conducted by Arriola-Villalobos et al. [16] in 2012 examined a smaller population (*n* = 19) but offered the longest follow-up for this combined procedure currently published in literature. At a mean follow-up of 53 months, a significant reduction in mean IOP of 3.16 mmHg was still demonstrated from a baseline of 19.4 mmHg (16.3% reduction, *P* = 0.002). While all subjects were using at least 1 IOP lowering medication at baseline, by the end of follow-up 8 subjects (42.1%) still did not require any hypotensive medication. Although limited by being an uncontrolled study with a small sample size, these results suggest that the efficacy of iStent implantation may persist in the long term.

The most recent published data on the long-term efficacy of combined cataract and iStent implantation demonstrated outcomes very similar to previous studies. In one study with a 3-year follow-up, iStent placement achieved an IOP reduction from 24.1 ± 6.9 mmHg at the baseline to 14.9 ± 2.3 mmHg, with use of glaucoma medications eliminated in 74% of patients [17]. Fea et al. also published long-term results, including a comparison between cataract extraction as a solo procedure and cataract extraction combined with implantation of one stent. Although not statistically significant, the microbypass stent combined with phacoemulsification group demonstrated a consistently reduced IOP throughout the entire study period, starting from 17.8 ± 2.7 mmHg at baseline to 16.1 ± 2.0 mmHg at 12 months and finally to 15.9 ± 2.3 mmHg at 48 months. At long-term follow-up after washout, IOP in the group receiving phacoemulsification alone was significantly greater than that at baseline (20.4 ± 3.2  versus 16.7 ± 3.0 mmHg, *P* = 0.002) and a 14.2% difference in IOP compared to the combined group was reported, which was statistically significant (17.5 ± 2.3 mmHg in the combined group versus 20.4 ± 3.2 mmHg in the control group, *P* = 0.02) [18].

The largest prospective, randomized, controlled trial to be performed on this topic as of yet is a multicenter study conducted by the US iStent Study Group [15]. The study enrolled a total of 240 eyes with cataract and OAG which were randomized into 2 groups to receive either phacoemulsification alone (*n* = 123) or phacoemulsification combined with a single iStent (*n* = 117). In 2011 Samuelson et al. [15] published results from a 12-month follow-up, with the primary efficacy measure defined as an unmedicated IOP ≤21. The iStent group performed significantly better than the group receiving phacoemulsification alone, with 72% reaching the desired outcome of an IOP of <22 mmHg without glaucoma medications in the iStent group compared to 50% in the control group (*P* < 0.001). A secondary efficacy measure specified as an IOP reduction ≥20% without medication resulted in a similarly significant outcome, demonstrating 18% treatment difference between subjects receiving an iStent group and those receiving phacoemulsification alone (66% versus 48%, *P* = 0.003). While the mean reduction in IOP was similar in both groups at 12 months (as expected due to the study protocol calling for active management of IOP with medication), the iStent group subjects were able to achieve their IOP reduction with significantly fewer medications. The time to first medication was significantly longer in the iStent group, with control subjects taking more ocular hypotensive medications at 1 week compared to iStent group subjects at 1 year. At 12 months the mean decrease in medications from baseline was larger in the iStent group (1.4 versus 1.0, *P* = 0.005) and fewer subjects in the iStent group required IOP lowering medication compared to those in the control group (15% versus 35%, *P* = 0.001). In 2012, Craven et al. [19] reported the data from a 24-month follow-up in the same study subjects. Although a difference in IOP lowering medication use between the 2 groups was no longer statistically significant (the study protocol was not sufficiently powered for a 2-year efficacy evaluation), a difference favoring the iStent group was still observed when considering the same primary efficacy outcome of an unmedicated IOP ≤21 (*P* = 0.036).

A meta-analysis comparing iStent implantation with phacoemulsification versus phacoemulsification alone has been recently published by Malvankar-Mehta et al. [11] in 2015. This meta-analysis reported that while both strategies caused reduction of IOP and the number of medications used in the long term, the combined iStent implantation had significantly better results. Phacoemulsification alone resulted in a 4% mean decrease in IOP from baseline; however, addition of an iStent increased this to a 9% reduction; concurrent implantation of 2 iStents additionally improved this to a 27% reduction in IOP. Moreover, combination surgery resulted in a weighted mean reduction in the number of glaucoma medications by 1.33 per patient, compared to 1.01 with phacoemulsification alone. Success of the combined surgery continued into the long term, with the meta-analysis finding a significant reduction of glaucoma medications by 12 months postoperatively, which remained significant until 4 years of follow-up. Despite significant heterogeneity between studies examined in the meta-analysis, the results concluded that the combined iStent with phacoemulsification surgery significantly outperforms phacoemulsification alone in terms of both IOP reduction and medication burden.

## 3. iStent as a Solo Procedure

While currently not performed commonly, the implantation of an iStent as a solo procedure has been advocated for by some authors. The earliest studies on this topic were prospective, interventional case series on patients with OAG published by Spiegel et al. in 2007 [7]. The study demonstrated a mean IOP reduction of 23.9% among the 6 patients examined in the study, from a baseline of 20.2 ± 6.3 mmHg to 15.3 ± 3.7 mmHg. Buchacra et al. [20] published results from a similar case series in 2011 which examined 10 patients with secondary OAG (including traumatic, steroid induced, pseudoexfoliative, and pigmentary glaucoma) who underwent single iStent implantation without cataract surgery. Of the 10 patients enrolled in this study, 7 had phakic lenses. The surgery was found to be effective, with 8 patients averaging a 27.3% reduction in IOP after 12 months. Additionally, the solo procedure proved to be very safe, with no complications reported among phakic eyes.

A prospective, nonrandomized study conducted by Ahmed et al. [21] examined the efficacy of 2 iStents implanted simultaneously in a solo procedure (Figure 1(c)). The study enrolled 39 phakic subjects with OAG which were on 2 IOP lowering medications preoperatively. Following a washout of all medications, subjects received iStent implantation surgery and were concurrently started on travoprost topical medication. From a mean baseline prewashout IOP of 22.2 ± 2.0 mmHg, IOP was reduced to 14.0 ± 2.2 mmHg by 1 month and 13.0 ± 2.4 by 12 months, with reduction of 1 medication. At the 12-month follow-up, all 39 subjects had achieved an IOP ≤18 mmHg and a reduction ≥20% from baseline solely on travoprost. Moreover, 29 patients (74.4%) achieved an IOP reduction ≥40% from baseline. Following a washout leading up to a 13-month follow-up, it was observed that the mean unmedicated IOP decreased from 25.3 ± 1.8 mmHg preoperatively to 17.1 ± 2.2 mmHg, with an IOP reduction of 8.2 mmHg or 32.4%. The results suggest that iStent implantation could serve as a substitute for patients using multiple ocular hypotensive medications. Results from a 5-year follow-up on the same subjects are pending and may help elucidate long-term effects of solo iStent implantation.

One control trial has been conducted examining the potential for increased efficacy with the addition of a third iStent in a solo procedure. Conducted by Belovay et al. in 2012 [22], the study compared the use of 1, 2, or 3 iStents, with 30 patients enrolled in each of the 3 groups. It showed that the group that simultaneously received 3 iStents presented with a mean IOP of 12.9 ± 1.6 mmHg at 6 months postoperatively, compared to a baseline of 24.3 ± 3.7 mmHg, with a reduction of 41%. While this outperformed the single iStent group, which experienced a mean IOP reduction of 31%, it performed similarly to the 2 iStents group which had an identical IOP reduction of 41%.

A prospective pilot study evaluated the solo implantation of 2 iStents in 39 patients using one topical IOP lowering medication prior to washout. The primary end point was IOP reduction ≥20% without medication compared to baseline unmedicated IOP at 12-month follow-up and a secondary end point of IOP ≤18 mmHg without medication at 12-month follow-up. The primary and secondary efficacy end points were each achieved by 92.3% of subjects (*n* = 36; 95% CI: 79.1%, 98.4%). Additionally, mean reduction in IOP from baseline was 44%. Most subjects maintained these target IOP thresholds through month 36, with an IOP reduction ≥20% achieved by 86.2% (*n* = 25; 95% CI: 68.3%, 96.1%) and IOP ≤18 mmHg achieved by 89.7% of patients (*n* = 26; 95% CI: 72.6%, 97.8%) [23].

Katz et al. also evaluated the efficacy and safety of the implantation of 1 or multiple iStents as a solo procedure. Subjects were submitted to washout and divided into groups to receive either 1 (*n* = 38), 2 (*n* = 41), or 3 (*n* = 40) stents. The same primary efficacy end point of an IOP reduction ≥20% without medication at 12 months compared to baseline unmedicated IOP and secondary end point of IOP ≤18 mmHg without medication at 12 months were used in this study. Additional measures included a proportional analysis of subjects with IOP ≤15 mmHg at 12 months. For all analyses of efficacy, patients could not be using topical ocular medication at 12 months and must not have undergone any additional surgical procedures for glaucoma by month 12.

Both the primary and secondary efficacy end points were achieved by 89.2% of one-stent, 90.2% of two-stent, and 92.1% of three-stent subjects. At 12-month follow-up, an IOP ≤15 mmHg without medication was achieved by 64.9% (*n* = 24, 95% CI: 47.5%–79.8%) of one-stent subjects, 85.4% (*n* = 35, 95% CI: 70.8%–94.4%) of two-stent subjects, and 92.1% (*n* = 35, 95% CI: 78.6%–98.3%) of three-stent subjects. Following a 1-month medication washout period at month 12 for eyes on medication, mean unmedicated IOP at months 12–13 were 14.9 ± 1.9 mmHg, 13.6 ± 2.1 mmHg, and 12.7 ± 2.1 mmHg in the three respective groups. IOP reduction was sustained in each of the groups throughout the 18-month postoperative period, with a greater reduction observed in the multiple-stent groups versus the one-stent group. At 18 months, mean IOP was 15.6 ± 1.5 mmHg in the one-stent group, 13.8 ± 1.3 mmHg in the two-stent group, and 12.1 ± 1.2 mmHg in the three-stent group [24].

A recent 2015 meta-analysis assessing solo iStent implantation, conducted by Malvankar-Mehta et al. [25], examined 5 studies with a total of 248 subjects for quantitative synthesis. Despite significant heterogeneity between studies, the meta-analysis concluded that a 22% weighted mean IOP reduction from baseline was observed at 18 months after 1 iStent was implanted, 30% weighted mean IOP reduction from baseline was observed at 6 months after 2 iStents were implanted, and 41% weighted mean IOP reduction from baseline was observed at 6 months after 3 iStents were implanted, with a statistically significant reduction found in all 3 groups. Additionally, a significant reduction in ocular hypotensive medication use was seen after implantation in all 3 groups, with a mean reduction of 1.2 bottles per patient at 18 months after 1 iStent was implanted, 1.45 bottles per patient at 6 months after 2 iStents were implanted, and 1 bottle per patient at 6 months after 3 iStents were implanted. Although data was limited by the fact that only a single study had examined the impact of 3 iStents, results suggested that the IOP decrease correlates positively with the number of iStents injected. Overall, the meta-analysis concluded that iStent implantation as a solo procedure is effective in lowering IOP and reducing dependency on topical glaucoma medications.

## 4. Complications of iStents

A key aspect of the iStent, as a MIGS device, is its favorable safety profile. Clinical trials and case series have consistently reported few to no adverse events following its implantation [10, 15, 19, 26]. Moreover, when complications occur they are often due to issues with the simultaneously performed cataract surgery rather than the result of issues with the iStent itself.

The randomized control trial for the iStent Study Group, reported on by Samuelson et al. [15] and Craven et al. [19], found that, among the 240 eyes enrolled, implantation of the iStent along with cataract surgery did not result in substantial additional risk or adverse events. The increased efficacy demonstrated in the iStent group during the 24-month study was achieved with no compromise in visual outcomes and with a safety profile comparable to cataract surgery alone. The most common early postoperative complications found in the iStent group were related to iStent malpositioning and obstruction (by iris, blood, etc.). Incidence of these events in the iStent Study Group was 3% and 4%, respectively. A variety of options for managing these complications have proven to be safe and clinically viable, ranging from observation while waiting for spontaneous resolution to more aggressive options such as laser therapy, stent repositioning, or stent replacement. In the iStent Study Group, 5 subjects who received an iStent (4.5%) required secondary surgery to fix iStent related complications (3 stent repositionings, 1 stent replacement, and 1 laser iridoplasty). Importantly, there was no evidence of any severe adverse events following iStent implantation.

While reports of most complications are consistent across all studies, other studies have found a wide range in frequency of complications related to iStent malpositioning. Compared to the iStent Study Group results of a 3% incidence among subjects, a 2010 study by Fernández-Barrientos et al. [26] found that 6 of 34 (17.6%) iStents were malpositioned upon follow-up, and results from Fea in 2010 [10] showed that 2 of 12 (16.7%) were malpositioned. It is notable, however, that none of these cases led to any significant adverse events, nor did any require resurgery. It is likely that this variation among reported results is largely due to a lack of specific standardized criteria defining malpositioning, along with the absence of a universal protocol for determining if surgical intervention is necessary. Among studies examined in this review, surgical intervention (e.g., iStent repositioning or removal) or laser procedures were necessitated in 4.5% to 11.3% of study subjects that experienced complication related to iStent malpositioning and obstruction.

Hyphema is another complication seen in the early postoperative stage and is usually a consequence of blood reflux from the iStent. It is in fact a sign that indicates patency of the iStent and typically resolves spontaneously within 1 week postoperatively. Studies where hyphema was reported largely did not specify if it was a notable complication or normal reflux as a result of the procedure. Given this, the reported frequency of this complication varies greatly among studies from 2.3% to 70% [14, 20].

One rare complication occurs when an ophthalmologist cannot locate an implanted iStent under gonioscopy postoperatively, with one such example of this seen in a study by Fea et al. [27] published in 2014. Ichhpujani et al. [28] conducted a laboratory study in 2010 testing the efficacy of 3 different imaging technologies in their ability to locate a deliberately misplaced iStent. The results demonstrated that ultrasound biomicroscopy was able to locate the “missing” iStents with the best reliability, compared to optical coherence tomography and B-scan ultrasonography.

## 5. iStent* inject*

**Figure 2:**
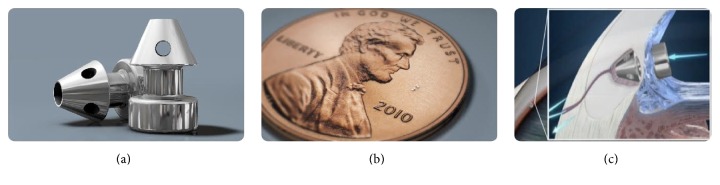
Illustration of the second-generation iStent* inject* (a); size comparison of the iStent* inject* (b); schematic illustration of iStent* inject* placement in trabecular meshwork (c). ((a)–(c), courtesy of Glaukos Corporation).

The newer, second generation of the iStent is a smaller version of the original model (Figures 2(a) and 2(b)), called the GTS-400 iStent* inject *(Glaukos, Laguna Hills, CA). Developed to reduce IOP in the same safe and effective way, the iStent* inject* is proposed to have an easier learning curve largely due to the device's completely different structure compared to the first generation, notably evidenced by the absence of the snorkel (Figures 2(a) and 2(c)). The new device also includes a modified injector that can be simultaneously loaded with 2 stents, an important improvement that allows surgeons to place both stents with a single entry into the eye. Bahler et al. [29] conducted a laboratory study with the new device utilizing human donor eyes, with a method similar to what has already been performed with first-generation iStent. The results demonstrated that addition of a second iStent significantly increased the outflow.

One of the first reports on the iStent inject was published in 2013 by Arriola-Villalobos et al. [30], in which 20 patients underwent combined phacoemulsification and implantation of 2 iStent* inject* stents as part of a prospective, uncontrolled, interventional case series study. At a 12-month follow-up, the mean washout baseline IOP of 26 ± 3.1 mmHg was decreased by 35.7% to 16.7 ± 2.2 mmHg (9.4 ± 3 mmHg reduction, *P* < 0.001). Mean number of glaucoma medications also fell from 1.3 ± 0.6 to 0.3 ± 0.5 (*P* < 0.001), with 75% patients still completely off medication at 1 year. The study observed no adverse events and concluded that combined cataract surgery with implantation of 2 iStent* inject* stents seems to be a safe and effective procedure.

In 2014, Voskanyan et al. [31] presented results from a prospective, multicenter, open-label study on the implantation of 2 iStent* inject* stents as a solo procedure in 99 phakic and pseudophakic subjects with OAG. At a 12-month follow-up, mean baseline washout IOP values decreased by 10.2 mmHg (39.7% reduction) from 26.3 ± 3.5 to 15.7 ± 3.7 mmHg. Additionally, 66% of subjects had an IOP ≤18 mmHg without medication, and 81% achieved an IOP ≤18 mmHg with either 1 or no medications. Medication burden also improved in 86.9% of subjects, with 15.2% experiencing a reduction of 1 medication and 71.7% discontinuing use of 2 or more medications postoperatively.

Fea et al. [27] conducted a prospective, multicenter, randomized clinical trial in 6 countries, enrolling OAG patients with uncontrolled IOP on 1 medication who either underwent implantation of 2 iStent* inject* stents or received medical therapy consisting of a fixed combination. Ninety-four patients were enrolled in this study for the iStent group, the majority of whom were phakic (98%) and Caucasian (100%). After 12 months of follow up, 94.7% of the eyes in the iStent group reported an IOP reduction ≥20% without use of any medications. The mean baseline IOP after washout was 25.2 ± 1.4 mmHg, and after 12 months the mean IOP decreased to 13.0 ± 2.3 mmHg. A favorable safety profile was achieved in the iStent group as measured by a stable best corrected visual acuity and cup-to-disk ratio among subjects throughout the study, as well as few adverse events.

Complication rates with the iStent* inject* have been found to be comparable to those from the previous model. It is notable that Klamann et al. [32] showed that blood reflux occurred in 91% of the surgeries involving the iStent* inject*; however, there was no incidence of complicated hemorrhage.

## 6. Cost Considerations

There is a lack of studies considering the cost effectiveness of the iStent; there exists only 1 published study to date on this topic [8]. The study, based in Canada, demonstrated that implantation of 2 iStents could possibly reduce the costs of the glaucoma treatment in comparison to the use of topical medications. Over a 6-year period, potential savings were estimated to be CA$1272 when comparing the iStent to a drug regimen using 2 medications and CA$2124 when compared to 3 medications. The study was limited by solely considering the placement of 2 simultaneous iStents, a relatively uncommon practice that is not approved in some countries (e.g., United States). Further limitations arose from difficulty predicting the effective duration at which iStents can continue to control IOP due to the lack of randomized controlled trials with long-term follow-up times. It is also important to be aware of variation in cost of medications, surgery fees, and many other variables like the cost of the device itself. The financial viability of the iStent device will play a crucial role for patients and doctors when deciding to implement this technology, making it critical for more studies to be conducted on this topic.

## 7. Conclusion

The considerable focus and interest of the medical community with MIGS in the last decade demonstrates that ophthalmologists are anxious for advancements in the surgical treatment of glaucoma. It is clear that single trabecular microbypass stents do not have an IOP reducing power comparable to more invasive surgeries such as trabeculectomy or tube shunt surgery. However, it is important to understand that the iStent is not intended to totally replace these procedures. MIGS, such as the iStent, instead have the potential to be a valuable option for glaucoma surgeons due to their precise indications, consistent efficacy, and ability to increase patient prognosis and quality of life. Additional high quality randomized controlled trials are still needed to confirm the advantages of MIGS over cataract surgery alone [33].

The favorable safety profile consistently demonstrated across studies is one of the key features of the iStent, as the potential for serious adverse events can be a significant deterrent for patients and physicians when considering surgical interventions during the initial and moderate stages of glaucoma. New devices are continually being developed and improved, and current MIGS devices are likely only the beginning of a new era in glaucoma management. As valuable experience is gained performing ab interno MIGS, increasing familiarity with angle anatomy and iStent placement, and as newer stent designs are developed, there is promise of continual improvement in the surgical management of glaucoma.

## Figures and Tables

**Table 1:** Summary of iStent randomized controlled trials.

Authors (year)	TG (*n*)	CG (*n*)	Device	Procedure	TG mean IOP reduction (%)	CG mean IOP reduction (%)	TG med. reduction (%)^*∗*^	CG med. reduction (%)^*∗*^	Follow-up (months)
Samuelson et al. [15] (2011)	117	123	iStent	Phaco. versus Phaco. + 1 iStent	8.2	5.4	86.7	73.3	12
Craven et al. [19] (2012)	117	123	iStent	Phaco. versus Phaco. + 1 iStent	8.1	4.3	80.0	66.7	24
Fernández-Barrientos et al. [26] (2010)	17	16	iStent	Phaco. versus Phaco. + 2 iStents	27.3	16.5	100	41.7	12
Fea [10] (2010)	12	24	iStent	Phaco. versus Phaco. + 1 iStent	17.3	9.2	80.0	31.6	15
Fea et al. [27] (2014)	94	98	iStent inject	2 iStents versus med.	38.4	36.2	N/A	N/A	12

CG, control group; IOP, intraocular pressure; med, medication; Phaco., phacoemulsification; TG, treatment group.

^*∗*^The numerical value listed under “med. reduction” represents the decrease in mean number of IOP lowering medications used postoperatively.

**Table 2:** Summary of iStent case series.

Authors (year)	*n*	Procedure	Device	Mean IOP reduction (%)	Medication reduction (%)^*∗*^	Follow-up (months)
Spiegel et al. [7]^1^ (2007)	6	1 iStent	iStent	23.9	18.5	12
Buchacra et al. [20]^1^ (2011)	10	1 iStent	iStent	27.3	62.0	12
Ahmed et al. [21]^1^ (2014)	39	2 iStents + travoprost	iStent	46.9	50	18
Voskanyan et al. [31]^1^ (2014)	99	2 iStents	iStent inject	39.7	N/A	12
Arriola-Villalobos et al. [30]^1^ (2013)	20	Phaco. + 1 iStent or 2 iStents	iStent inject	35.7	76.9	12
Spiegel et al. [13]^1^ (2008)	47	Phaco. + 1 iStent	iStent	25.4	66.7	6
Spiegel et al. [12]^1^ (2009)	47	Phaco. + 1 iStent	iStent	21.4	75.0	12
Arriola-Villalobos et al. [16]^1^ (2012)	19	Phaco. + 1 iStent	iStent	16.3	63.6	60
Patel et al. [14]^1^ (2013)	44	Phaco. + 1 iStent	iStent	20.9	74.3	6
Belovay et al. [22]^1^ (2012)	53	Phaco. + iStents (2 or 3)	iStent	20.2–20.4	64.3–84.6	12
Klamann et al. [32]^2^ (2015)	35	1 iStent in phakic OAG	iStent inject	33–35	N/A^3^	6
El Wardani et al. [34]^2^ (2015)	131	Phaco. alone and 1 iStent or 2 iStents	iStent	N/A^4^	27^5^	6

IOP, intraocular pressure; *n*, number of eyes enrolled; Phaco., phacoemulsification; OAG, open-angle glaucoma.

^*∗*^The numerical value listed under “medication reduction” represents the decrease in mean number of IOP lowering medications used postoperatively.

^1^Prospective studies.

^2^Retrospective studies.

^3^No statically significant difference was found at the end of follow-up.

^4^Percentage of reduction was not available in abstract (epub ahead of printing).

^5^Number of medications was reduced by 8% in the phacoemulsification alone group, 27% when using one iStent, and 45% when using 2 iStents.
